# Spectral domain optical coherence tomography as an adjunctive tool for screening Behçet uveitis

**DOI:** 10.1371/journal.pone.0208254

**Published:** 2018-12-11

**Authors:** Hae Min Kang, Hyoung Jun Koh, Sung Chul Lee

**Affiliations:** 1 Department of Ophthalmology, Catholic Kwandong University College of Medicine, International St. Mary’s Hospital, Incheon, Republic of Korea; 2 Institute of Vision Research, Department of Ophthalmology, Yonsei University College of Medicine, Seoul, Republic of Korea; Charite Universitatsmedizin Berlin, GERMANY

## Abstract

**Background:**

This study investigated the association of central macular thickness (CMT) and macular volume (MV) with severity of Behçet uveitis in the absence of macular edema (ME).

**Methods:**

This retrospective, interventional study included a total 131 treatment-naïve Behçet patients with varying degree of uveitis in the absence of ME. The mean CMT and MV were obtained by spectral domain optical coherence tomography (SD ODT). The patients were classified according to the anatomical classification of Behçet uveitis. The main outcome measure was comparison of mean CMT and MV with the types of Behçet uveitis.

**Results:**

Sixty patients (45.8%) with no uveitis, 41 patients (31.3%) with anterior uveitis, 18 patients (13.7%) with posterior uveitis, and 12 patients (9.2%) with panuveitis. The mean CMT were 261.6±22.2 μm in no uveitis, 268.1±17.8 μm in anterior uveitis, 306.4±32.9 μm in posterior uveitis, and 300.4±44.0 μm in panuveitis (*P < 0*.*001*). The mean MV was 8.7±0.3 mm^3^ in those without uveitis, 8.8±0.3 mm^3^ in anterior uveitis, 9.9±1.1 mm^3^ in those with posterior uveitis, and 9.7±0.4 mm^3^ in panuveitis (*P < 0*.*001*). Types of Behçet uveitis was the only significant factor correlated with the mean CMT (B = 18.170, β = 0.408, P < 0.001) and the mean MV (B = 0.328, β = 0.652, P < 0.001).

**Conclusions:**

The mean CMT and MV were significantly thicker in the Behçet uveitis with posterior involvement. SD OCT can be used for an adjunctive tool for screening Behçet uveitis, especially for the presence of posterior involvement.

## Introduction

Behçet’s disease is a chronic, systemic disease characterizing immune-mediated vasculitis [1,2]. Behçet’s disease involves mucous membranes, skin, eye, gastrointestinal tract, joints, vessels, and neurologic systems [1,2]. Endemic area includes the Middle East, the Mediterranean regions, and the Central and East Asia such as Korea [2,3]. In Korea, the overall prevalence of Behçet’s disease is approximately 35.0 per 100,000 population, with gradual increase of prevalence [4]. The expected prevalence of Behçet’s disease is estimated as 36.9 to 44.7 per 100,000 population between 2016 and 2025 [4].

Ocular involvement occurs in approximately 60 to 80% of the patients with Behçet’s disease, and posterior involvement has been reported in 50 to 93% of those with ocular involvement [1,5–7]. Retinal vasculitis, which represents the essential finding in Behçet’s disease, is characterized by an occlusive, necrotizing vasculitis of posterior pole [1,7]. Ocular involvement, especially posterior involvement is the most serious complication because it can lead to permanent visual deterioration [1,7–10]. Thus, screening for Behçet uveitis is important when managing the patients with Behçet’s disease.

Among the various ophthalmologic evaluation methods, fluorescein angiography (FA) is the standard tool for the detection and treatment monitoring of the patients with Behçet’s disease, especially for posterior involvement [11,12]. Diffuse vascular leakage, diffuse macular leakage, and disc leakage are predominant FA findings of posterior involvement in Behçet uveitis [13]. FA is also clinically important because fluorescein leakage from retinal vessels may be observed before the development of obvious ophthalmoscopic findings of posterior uveitis [7]. However, repeated use of FA is somewhat limited because of its invasiveness. Currently, advanced optical coherence tomography (OCT) such as spectral domain OCT (SD OCT) is the standard method for detecting macular complications in patients with uveitis [14–16]. SD OCT is useful for detecting, monitoring treatment outcomes, and prognoses for the visual outcomes of ME in patients with uveitis [15–19]. OCT can detect ERM and its risk factors in patients with uveitis [20], and OCT with enhanced depth imaging (EDI) modality has enabled investigations to determine the choroidal thickness in patients with uveitis. Several studies have suggested that choroidal thickness is correlated with the activity of the uveitis and can be used as a monitoring tool for disease progression [21–25]. These various studies have increased the overall utilization of OCT in patients with uveitis.

A few studies have investigated the macular thickness (MT) by SD OCT in patients with uveitis without macular complications [26–28]. These studies showed that MT was significantly thicker in eyes with uveitis than in non-affected contralateral eyes for all forms of uveitis. One study suggested that the normal-appearing thicker retina of the eyes of patients with uveitis may represent a precursor state of ME, and recommended careful monitoring using SD OCT [26]. Other studies suggested that the determination of MT by OCT can be used as a monitoring tool for the disease activity of uveitis [27,28]. Overall, these studies suggest that close monitoring of MT by OCT may be helpful in managing patients with uveitis [26–28]. However, these studies did not investigate the association of MT with the anatomical involvement of uveitis, especially in the presence of posterior uveitis, and also disease entities were mixed.

In this current study, we investigated if SD OCT can be used as an adjunctive tool for screening Behçet uveitis. We investigated the central MT (CMT) and the macular volume (MV) by using SD OCT in the patients with Behçet disease, and compared those parameters according to the types of Behçet uveitis.

## Methods

### Enrollment of study subjects

This study was a retrospective, observational study performed at the Catholic Kwandong University College of Medicine, International St. Mary’s Hospital. We retrospectively reviewed the medical records of the patients with treatment-naïve patients with Behçet’s disease who underwent ophthalmologic screening for Behçet uveitis. This study was approved by the Institutional Review Board of International St. Mary’s Hospital, Catholic Kwandong University College of Medicine, and adhered to the tenets of the Declaration of Helsinki. Because of the retrospective manner of this study, informed consent for each patient was waived by the Institutional Review Board.

For definitions used in this study, uveitis was defined according to the classification of the Standardization of Uveitis Nomenclature (SUN) working group [29]. For Behçet’s disease, the revised Simizu’s criteria was used for the diagnosis [30].

For the study, the treatment-naïve patients for both Behçet’s disease and Behçet uveitis were included with preserved foveal contour and without any intraretinal/subretinal fluid on SD OCT. Exclusion criteria were as follows: 1) eyes with macular complications such ERM documented by SD OCT; 2) eyes with severe media opacities, such as severe lens opacity, obscuring clear SD OCT imaging of the retina; 3) those with other concomitant ocular diseases with retinal vascular involvement, such as diabetic retinopathy, and 4) pathologic myopia due to segmentation errors in SD OCT images.

The primary outcome measure was the use of SD OCT to determine any difference of the mean CMT and the mean MV according to the types of Behçet uveitis.

### Ocular examination

At the first clinic visit, a full baseline examination, including a slit lamp examination, an intraocular pressure measurement using a non-contact tonometer, and a detailed fundus examination after dilation, was performed. Refractive error for each eye was measured using an autorefractor and then converted to spherical equivalents in diopters (D). FA was performed using the Heidelberg Retina Angiograph system (HRA-2; Heidelberg Engineering, Heidelberg, Germany) with a confocal scanning laser ophthalmoscope. SD OCT (Spectralis; Heidelberg Engineering) with an EDI modality was used for evaluation of the macular thickness. Images were acquired using horizontal raster pattern scans, which were obtained via a 30° × 15° scan field, consisting of 20 sections. The MV for each eye was automatically calculated by the embedded program in the SD OCT, and used for analyses. The CMT was defined as a mean retinal thickness in the central subfield, a region with a diameter of 1.0 mm around the fovea. The inner and the outer rings had diameters of 3.0 mm and 6.0 mm, respectively. All SD OCT images were reviewed and checked for any possibility of segmentation errors.

### Statistical analysis

Data were presented as the mean ± standard deviation, unless otherwise indicated. Baseline characteristics included age, sex, refractive error, number of cells, degrees of vitreous haze, CMT, and MV. For comparison, data from the right eye of each patient was used if the uveitis severity was equivalent between both eyes. If the severity of uveitis was different, the clinical data from the eye with more severe uveitis was used for the comparison. IBM SPSS Statistics software for Windows, version 22.0 (IBM Corporation, Somers, NY, USA) was used for statistical analyses. For comparison among the multiple groups, Kruskal Wallis test was used for continuous variables and the chi square test was used for the analyses of categorical variables. For comparison between two groups according to the posterior involvement, the Student’s *t*-test was used for the analyses of continuous variables and the chi square test was used for the analyses of categorical variables. The comparison of CMT and MV after treatment for posterior uveitis or panuveitis, repeated measured analysis of variance (ANOVA) was used. Stepwise multiple regression analyses were performed to determine the associations of baseline factors with the mean CMT and the mean MV, respectively. Results with P < 0.05 were considered statistically significant.

## Results

### Baseline characteristics of the study population

A total 131 patients with Behçet’s disease were included in this study. Among the patients, 48 patients (36.6%) were male, and the mean age was 45.1±11.5 (range, 20–56 years). Among the 131 eyes used as interpretation, 110 eyes (84.0%) were the right eyes. The mean refractive errors were -1.3±2.2 (-4.5 diopters to 2.0 diopters). At the time of ophthalmologic evaluation, 60 patients (45.8%) had no evidence of uveitis, 41 patients (31.3%) did anterior uveitis, 18 patients (13.7%) did posterior uveitis, and 12 patients (9.2%) did panuveitis. None of the study population had intermediate uveitis.

### Comparison of the mean central macular thickness and the mean macular volume according to the types of Behçet uveitis

As for the absence of intermediate uveitis in our study population, the mean CMT and the mean MV were compared among the four types of Behçet uveitis (none, anterior uveitis, posterior uveitis, and panuveitis). The baseline characteristics are shown in Table 1.

**Table 1:** Baseline characteristics of the 131 patients with Behçet’s disease.

	None (N = 60, 45.8%)	Anterior uveitis (N = 41, 31.3%)	Posterior uveitis (N = 18, 13.7%)	Panuveitis (N = 12, 9.2%)	P value
Mean age (years)	44.4±12.1	44.8±10.4	47.2±13.2	46.8±11.3	0.788†
Sex (male/female)	16 (26.7%) /44 (73.3%)	12(29.3%) /29(70.7%)	13(72.2%) /5 (27.8%)	7 (58.3%) /5 (41.7%)	0.001*
Mean refractive errors (diopters, D)	-1.4±2.4	-1.2±2.0	-1.8±2.2	-1.4±1.2	0.374†
Mean grade of anterior chamber cells	0	1.1±0.8	0	2.6±1.1	<0.001†
Mean grade of vitreous haze	0	0	0.4±0.6	0.8±0.7	<0.001†

^†^ Kruskal Wallis test was used for continuous variables and

* chi square test was used for the analyses of categorical variables.

P < 0.05 were considered statistically significant.

In the patients with no evidence of uveitis, the mean CMT were 261.6±22.2 μm (231.0 to 297.0 μm; median 259.0 μm). The mean CMT was 268.1±17.8 μm (239.0 to 317.0 μm; median 268.0 μm) in anterior uveitis, 306.4±32.9 μm (258.0 to 360.0 μm; median 304.5 μm) in posterior uveitis, and 300.4±44.0 μm (268.0 to 415.0 μm; median 298.5 μm) in panuveitis (*P<0*.*001*).

**Fig 1:**
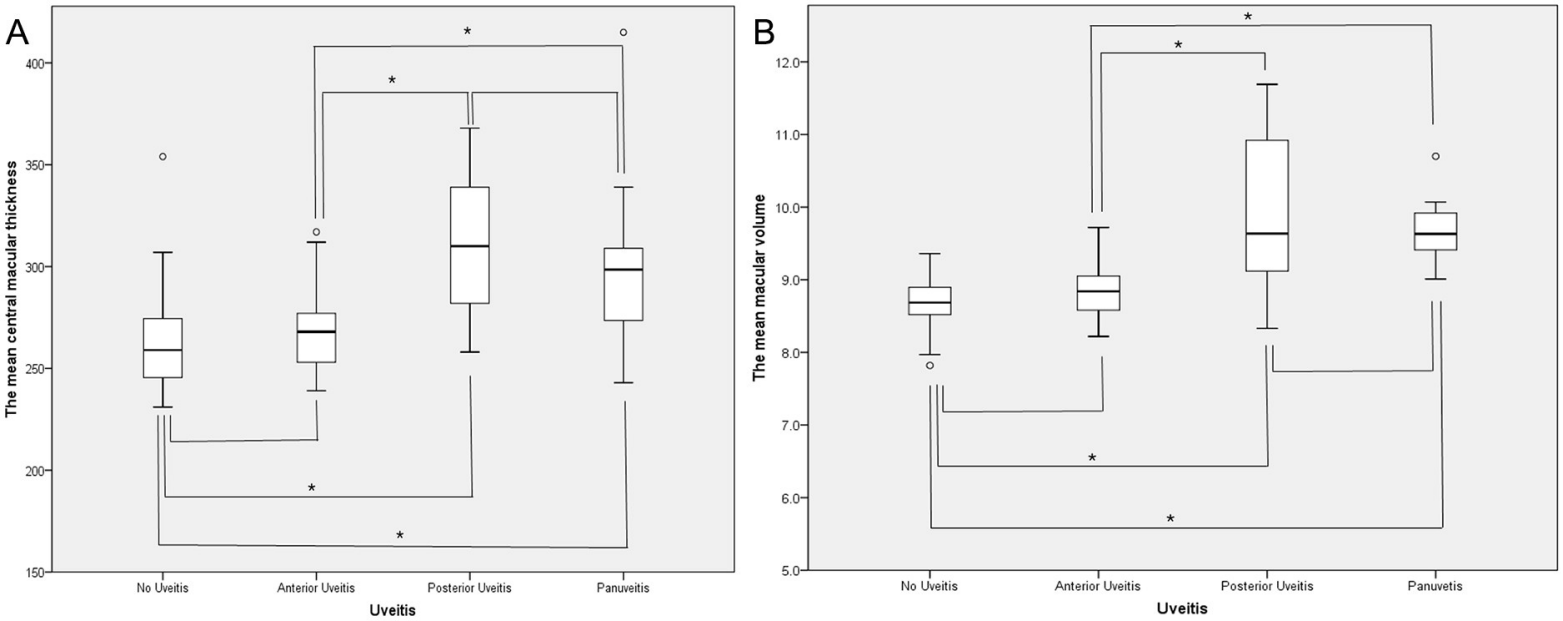
Subgroup comparison among the patients with Behçet disease. (A) Pairwise comparison of the mean central macular thickness (CMT, μm) showed that there was no significant difference in the mean CMT between those without uveitis and those with anterior uveitis (*P = 0*.*186*), and between those with posterior uveitis and those with panuveitis (*P = 0*.*851)*. However, the mean CMT was significantly thicker in those with posterior uveitis (*P<0*.*001*) and those with panuveitis (*P<0*.*0*01) than those without uveitis. Those with anterior uveitis showed similar tendency when compared with those with posterior uveitis (*P<0*.*001*) and those with panuveitis (*P<0*.*001*). (B) Pairwise comparison of the mean macular volume (MV, mm^3^) showed that there was no significant difference in the mean MV between those without uveitis and those with anterior uveitis (*P = 0*.*301*). and between those with posterior uveitis and those with panuveitis (*P = 0*.*200)*. However, the mean MV was significantly larger in those with posterior uveitis (*P<0*.*001*) and those with panuveitis (*P<0*.*0*01) than those without uveitis. Those with anterior uveitis showed similar tendency when compared with those with posterior uveitis (*P<0*.*001*) and those with panuveitis (*P = 0*.*006*). P value < 0.05 was marked as asterisk (*) in the figures.

The mean MV was 8.7±0.3 mm^3^ (7.8 to 9.4 mm^3^; median 8.7 mm^3^) in those without uveitis, 8.8±0.3 mm^3^ (8.2 to 9.3 mm^3^; median 8.8 mm^3^) in anterior uveitis, 9.9±1.1 mm^3^ (9.1 to 11.7 mm^3^; median 9.5 mm^3^) in those with posterior uveitis, and 9.7±0.4 mm^3^ (9.1 to 10.7 mm^3^; median 9.6 mm^3^) in panuveitis (*P<0*.*001*). However, subgroup analysis showed there was no significant difference in the mean CMT (*P = 0*.*186*) and the mean MV (*P = 0*.*301*) between those with no uveitis and those with anterior uveitis. Similarly, the mean CMT and the mean MV were not significantly different between those with posterior uveitis and those with panuveitis (*P = 0*.*851* and *P = 0*.*200*, respectively). Further detailed comparison among the patients is shown in Fig 1.

### Comparison of the mean central macular thickness and the mean macular volume according to the posterior involvement of Behçet uveitis

We the further classified the study population according to the posterior involvement of Behçet uveitis. Thus, those with no uveitis and anterior uveitis were classified as no posterior involvement (101 patients, 77.1%), and those with posterior uveitis and panuveitis were grouped as posterior involvement (30 patients, 22.9%). The mean grade of cells in the anterior chamber was 0.5±0.8 (0 to 4) in those without posterior involvement, and 1.0±1.5 (0 to 4) in those with posterior involvement (*P = 0*.*007*). The mean grade of vitreous haze was 0 in those without posterior involvement, and 0.6±0.7 (0 to 2) in those with posterior involvement (*P<0*.*001*).

The mean CMT was 264.5±20.7 μm (231.0 to 317.0 μm; median 264.0 μm) in those without posterior involvement, and 330.0±36.3 μm (268.0 to 415.0 μm; median 302.5 μm) in those with posterior involvement (*P < 0*.*001*). The mean MV was 8.7±0.3 mm^3^ (7.8 to 9.3 mm^3^; median 8.74 mm^3^) in those without posterior involvement, and 9.8±0.9 mm^3^ (9.1 to 11.7 mm^3^; median 9.6 mm^3^) (*P<0*.*001*).

### Changes of the mean central macular thickness and the mean macular volume after treatment for Behçet uveitis with posterior involvement

**Fig 2:**
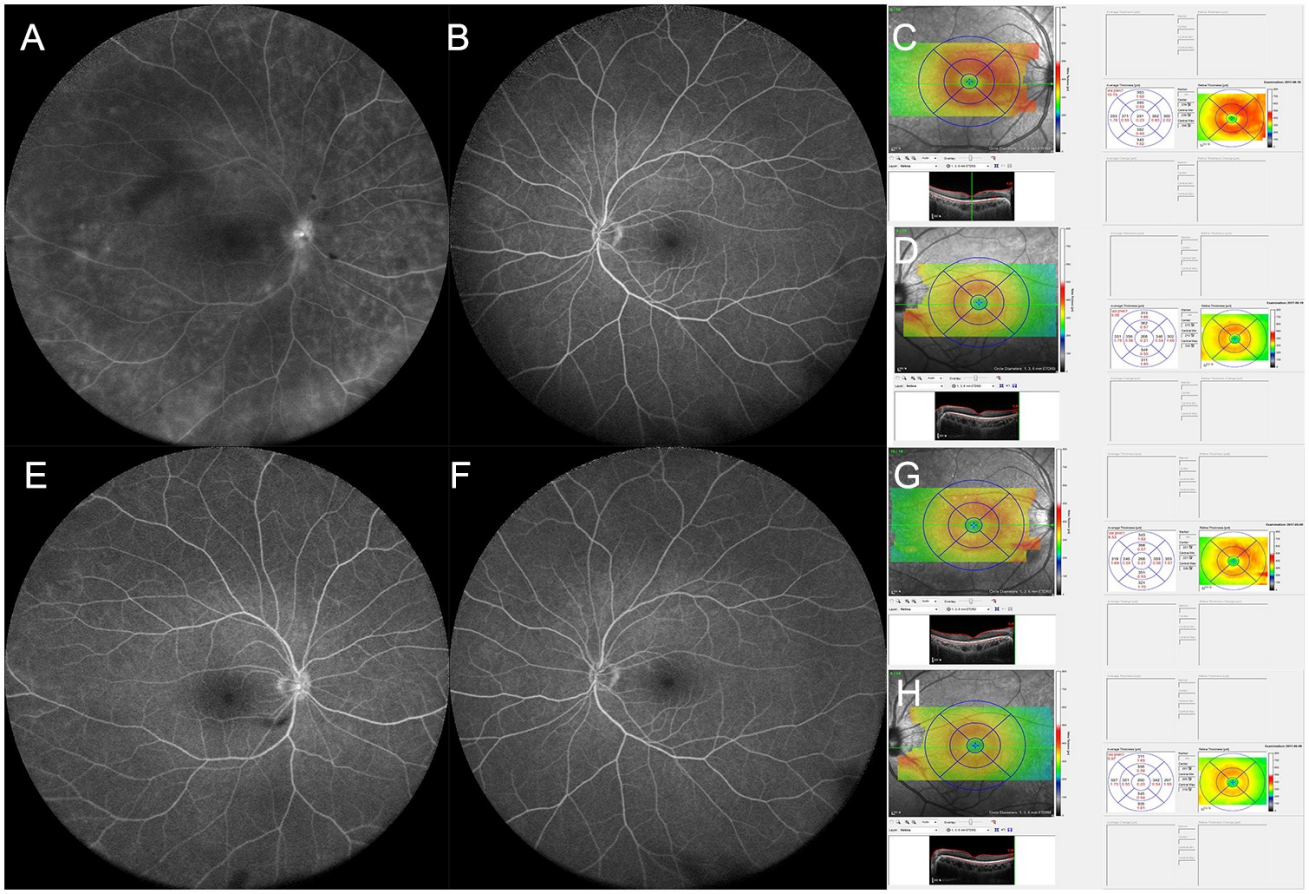
The representative case of Behçet uveitis in a 37-year-old male patient. A 37-year-old male patient with Behçet disease visited the ophthalmology clinic. Best-corrected visual acuity (BCVA) was 20/20 with Snellen visual acuity chart in both eyes, respectively. Slit lamp examination showed clear anterior chamber in both eyes. Detailed fundus examination showed vitreous haze 0.5+ in the right eye, and clear in the left eye. No remarkable retinal lesion was noted in both eyes. Fluorescein angiography (FA) showed (A) diffuse leakage with blocked fluorescence due to vitreous haze in the right eye, and (B) minimal leakage in the left eye at late phase. Spectral domain optical coherence tomography (SD OCT) showed (C) more macular thickening in the right eye than (D) the left eye. After intravitreal injection of dexamethasone implant (OZURDEX; Allergan, Irvine, CA, USA), BCVA was maintained as 20/20 with Snellen visual acuity chart in both eyes, and vitreous haze was resolved in the right eye. On FA, diffuse vascular leakage and blocked fluorescence were resolved in the right eye (E) and not changed in the left eye (F). On SD OCT, the macular thickness was improved in the right eye (G) and not significantly changed in the left eye (H).

**Fig 3:**
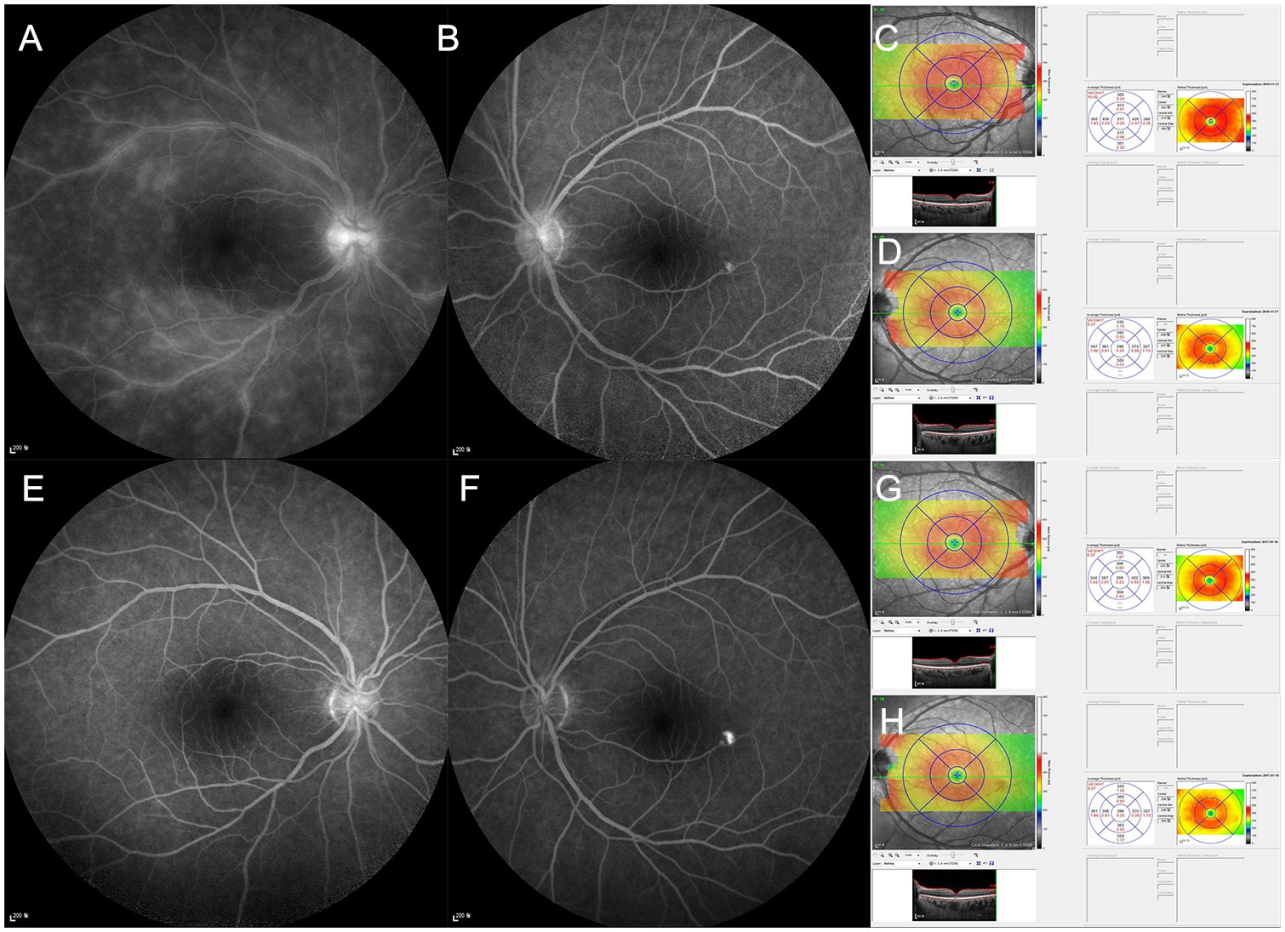
The representative case of Behçet uveitis in a 3*-year-old male patient. A 39-year-old male patient with Behçet disease visited the ophthalmology clinic. Best-corrected visual acuity (BCVA) was 20/20 with Snellen visual acuity chart in both eyes, respectively. Slit lamp examination showed clear anterior chamber in both eyes. Detailed fundus examination showed clear vitreous and not remarkable lesions in the fundus. Fluorescein angiography (FA) showed (A) diffuse vascular leakage with vessel wall staining, especially prominent in superior vascular arcade, in the right eye, and (B) minimal leakage in the left eye at late phase. Spectral domain optical coherence tomography (SD OCT) showed (C) more macular thickening in the right eye than (D) the left eye. After intravitreal injection of dexamethasone implant (OZURDEX; Allergan, Irvine, CA, USA), BCVA was maintained as 20/20 with Snellen visual acuity chart in both eyes. On FA, diffuse vascular leakage with vessel wall staining was resolved in the right eye (E) and not changed in the left eye (F). On SD OCT, the macular thickness was improved in the right eye (G) and not significantly changed in the left eye (H).

We investigated changes in the mean CMT and the mean MV after the treatment for Behçet uveitis with posterior involvement. Among the 30 patients, 24 patients underwent systemic steroid treatment and 4 patients did intravitreal injections of dexamethasone implant (OZURDEX; Allergan, Irvine, CA, USA; 4 patients), with mean follow-up period of 11.7±2.3 months (6–25 months). We compared the mean CMT and the mean MV at the time of diagnosis and at 2 to 3 months after the first treatment with treatment monitoring. At a mean of 2.2 months (2–3 months) after the first treatment, all patients showed improvement of retinal vascular leakage, and vitreous haze was significantly reduced from 0.7±0.8 (0.5~2) to 0.1±0.4 (0 to 0.5) (P<0.018). The mean CMT was significantly reduced from 330.0±36.3 μm (268.0 to 415.0 μm) to 280.2±30.0 μm (255.0–306.0 μm)(*P = 0*.*014*). The mean MV was also significantly decreased after treatment, from 9.8±0.9 mm^3^ (9.1 to 11.7 mm^3^) to 9.2±0.5 (8.1 to 10.4 mm^3^) (*P<0*.*001*). Representative figures are shown in Figs 2 and 3.

### Baseline factors correlated with the mean central macular thickness and the mean macular volume in Behçet uveitis

We investigated the possible factors associated with the mean CMT and the mean MV in Behçet uveitis by using stepwise multiple regression analyses. Baseline factors included age, sex, types of Behçet uveitis, the grade of anterior chamber cells, and the grade of vitreous haze. Among the baseline factors, types of Behçet uveitis was the only factor significantly correlated with the mean CMT (B = 18.170; β = 0.408; *P<0*.*001*) and the mean MV (B = 0.328, β = 0.652; *P<0*.*001*). Other factors were not significantly correlated with the mean CMT and the mean MV, shown in Table 2.

**Table 2:** Results of multiple regression analysis for the possible factors correlated with the mean central macular thickness and the mean macular volume in Behçet uveitis.

	The mean CMT		The mean MV	
	β	P value	β	P value
Sex	-0.002	0.834	0.016	0.828
Age	-0.018	0.985	-0.051	0.453
Types of uveitis	0.408	<0.001	0.652	<0.001
Refractive errors	-0.085	0.300	0.004	0.997
Grade of anterior chamber cells	0.119	0.095	-0.018	0.910
Grade of vitreous haze	-0.025	0.818	-0.115	0.180

## Discussion

In this study, we investigated if SD OCT can be used as an adjunctive tool for screening Behçet uveitis. We investigated the mean CMT and the mean MV in patients with Behçet’s disease by using SD OCT. In the absence of ME and other macular complications, the mean CMT and the mean MV were significantly increased in the patients with posterior involvement of Behçet uveitis than those without posterior involvement. After treatment for Behçet uveitis with posterior involvement, the mean CMT and the mean MV were significantly reduced from baseline in the patients. Subsequent analysis suggested that the mean CMT and the mean MV were significantly associated with the types of Behçet uveitis. Thus, our results suggest that SD OCT can be considered as an adjunctive tool for screening Behçet uveitis, especially for the posterior segment involvement.

ME in uveitis is the result of the breakdown of the blood-retinal barrier (BRB) [31], which is comprised of tight junctions between the endothelium of non-fenestrated capillaries and retinal pigment epithelium (RPE) cells. Pro-inflammatory cytokines such as TNF-α, IL-1, and TGF-β, as well as vascular endothelial growth factors are involved in the breakdown of the BRB at the level of the retinal capillary endothelium, which is the inner layer of the BRB [32–34]. Acute inflammatory conditions can therefore lead to damages of the outer layer of the BRB. The active transport mechanisms of the RPE maintain the adhesion between the photoreceptor cells and the RPE, so damage at the level of the RPE can lead to ME, even in the absence of an inner BRB breakdown [35–37]. In the patients with Behçet’s disease, the mean CMT and the mean MV were not significantly different between the patients without any uveitis and those with anterior uveitis. This suggests that anterior uveitis may not be associated with breakdown of the BRB significantly in Behçet’s disease. If subclinical inflammation exists even in the absence of obvious uveitis, our results suggest that it may not affect BRB as much as Behçet uveitis involving posterior segment. In our study, the mean CMT and the mean MV were significantly increased in the patients with posterior involvement of Behçet uveitis: both posterior uveitis and panuveitis. These findings suggest that posterior segment involvement of Behçet uveitis is associated with significant breakdown of the BRB, leading to generalized macular thickening and increased volume even in the absence of ME.

If SD OCT can be used as a screening tool for Behçet uveitis as shown in our study, it can provide qualitative data such as CMT and MV. The retinal thickness map provided by SD OCT is much easier for the acquisition, analyses, and comparison of data than conventional FA. Increased CMT and the mean MV on SD OCT in patients with Behçet uveitis may suggest posterior involvement of Behçet uveitis, and then further FA can be performed to confirm the disease status. This may help avoiding unnecessary FA in patients with Behçet uveitis. However, it does not mean that SD OCT can completely replace the role of FA in the patients with Behçet’s disease. SD OCT cannot provide the underlying retinal vascular status as well as detect retinal neovascularization. From our results, we suggest the expanded role of SD OCT in the patients with Behçet’s disease, not only for detecting macular complications such as ME and ERM, but also for screening Behçet uveitis, especially for the presence of posterior segment involvement.

In our study, we could also observe that the mean CMT and the mean MV was significantly reduced after treatment for Behçet uveitis involving posterior segment. This may suggest that SD OCT can be useful for treatment monitoring for Behçet uveitis. However, we only compared relatively short follow-up results in this study, which need further validation. Because Behcet uveitis is a chronic inflammatory disease, long-term vascular inflammation can result in permanent breakdown of BRB. In the presence of permanent vascular damage, the macula may be thickened to some extent regardless of disease activity. Then, the patients with chronic Behcet uveitis may show limited value of SD OCT in disease monitoring. To validate and widen the role of SD OCT for disease monitoring, further longitudinal study would be warranted for Behçet uveitis.

Our study has several limitations, including its retrospective nature and lacked patients with intermediate uveitis. We included treatment-naïve patients with Behcet’s disease, we could eliminate the possible effect of systemic diseases at the time of screening Behcet uveitis. However, this also resulted in relatively small number of the study population, which may limit the widespread use of our study findings. Thus, further prospective studies with larger study populations and longer follow-up period should be followed to support our findings in Behçet’s uveitis.

In conclusion, the mean CMT and the mean MV by SD OCT were significantly associated with the types of Behçet uveitis, especially in the presence of posterior involvement. Thus, the CMT and MV by SD OCT can be considered as an adjunctive tool for screening Behçet uveitis.
